# Case Report on Crossed Fused Renal Ectopia With a Large Calculus and Its Management

**DOI:** 10.7759/cureus.15512

**Published:** 2021-06-08

**Authors:** Qazi Kamran Amin, Sahal Arshad, Nouman Anthony, Zaland A Yousafzai, Sanan Arshad

**Affiliations:** 1 General Medicine, Rehman Medical Institute, Peshawar, PAK; 2 General Medicine, Rehman Medical Institue, Peshawar, PAK; 3 Medicine, Rehman Medical Institute, Peshawar, PAK; 4 General Internal Medicine, Hayatabad Medical Complex Peshawar, Peshawar, PAK

**Keywords:** ectopic kidney, renal caculus, crossed renal ectopia, dj stent, congenital anomalies of the kidney and urinary tract (cakut)

## Abstract

Among congenital renal anomalies, the ectopic kidney is a rare occurrence. Crossed fused renal ectopia (CFRE) - an even rarer subtype of ectopic kidney - is characterized by both kidneys being fused together on one side of the spine. CFRE is usually asymptomatic but can present with vague symptoms if the anomalous kidney becomes infected, is obstructed by calculus, or has a neoplastic change. There is no indication for surgical intervention if the kidney functions normally. This report presents a case of CFRE in a 31-year-old male with recurrent right flank pain resulting from a large calculus in the upper moiety of the fused kidney. The calculus was surgically removed by percutaneous nephrolithotomy (PCNL). The patient was discharged on analgesics, antibiotics, and potassium citrate tablets, with an order to follow up after one month. On follow-up, the patient’s double-J (DJ) stent was removed and an X-ray was performed to rule out any complications. No signs of stones, strictures, or other complications were noticed.

## Introduction

Ectopic kidney is a congenital renal anomaly characterized by the abnormal location of one or both kidneys. Ectopic kidney occurs in several forms (e.g., pelvic kidney, thoracic kidney, and crossed fused renal ectopia [CFRE]) [1]. CFRE, also known as a crossed dystopia, is a rare form of renal ectopia in which both kidneys are located on the same side of the spine. The estimated global incidence of CFRE is approximately 1 in 7,000 births [2].

CFRE is often asymptomatic but can rarely present with nonspecific or unrelatable symptoms, such as abdominal or flank pain, palpable mass, hematuria, and dysuria [3]. Due to its rarity and asymptomatic presentation, CFRE diagnosis is usually incidental and may be detected either by abdominal ultrasound or a kidney, ureter, and bladder (KUB) computed tomography (CT) scan. The most common complications of CFRE are obstruction, infection, neoplasia, and nephrolithiasis [4].

Primary treatment is seldom required for CFRE. However, because crossed fused kidneys have an anomalous blood supply, it is important to complete an angiogram before planning any surgical intervention to discern the kidneys’ exact location, blood supply, and relation to surrounding organs [5].

## Case presentation

**Figure 1:**
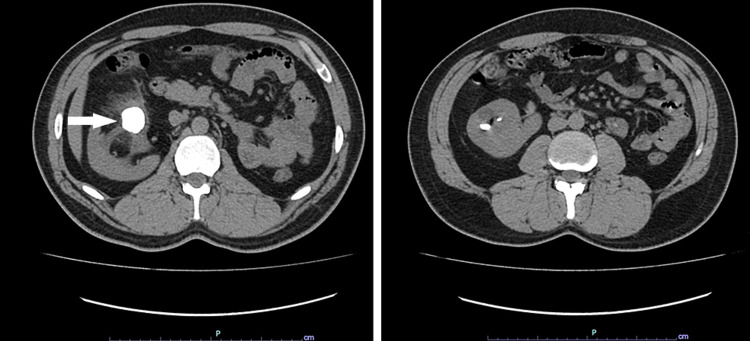
Computed tomography abdominal scan of a 31-year-old male showing right crossed fused renal ectopia with a large 2.7-cm calculus with fat stranding and moderate hydronephrosis.

A 31-year-old normotensive and normoglycemic male patient presented to us in the clinic with chief complaints of right flank pain associated with burning micturition and hematuria. The patient was a resident of Swabi, Pakistan, with good socioeconomic standing. On examination, the patient had moderate tenderness in the right flank region but was otherwise fit and well. The patient’s family history was unremarkable. His past medical history included similar episodes of right flank pain, first occurring in 2010, for which he was treated conservatively, and again in 2013 he underwent an ultrasound that showed a missing left kidney and a stone in the right kidney pelvis; however, no intervention was made at that time. In 2018, he experienced the same complaint of right flank pain; at that time surgical removal of the stone was advised but refused by the patient. In 2021, the patient’s symptoms worsened and he decided to undergo surgery. Laboratory investigations and routine urine examination showed a hemoglobin count of 14 g/dl; red blood cell count of 25-30 HPF; white blood cell count of 12-15 HPF; and 2+ proteinuria. Renal function tests showed a creatinine level of 1.0 mg/dl. A computed tomography (CT) urogram with contrast showed the right kidney measuring 17 cm in length and an absent left kidney (Figure 1).

**Figure 2:**
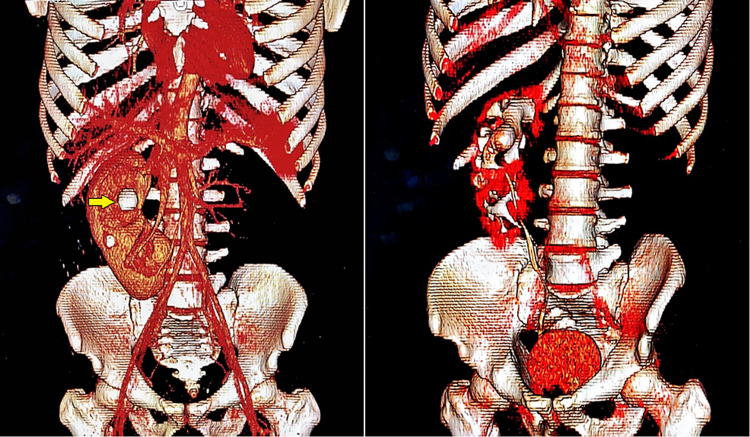
Three-dimensional reconstruction of a right crossed fused renal ectopia in a 31-year-old male. A large obstructive calculus can be seen in the upper calyx plus two smaller calculi in the lower calyx.

Three-dimensional reconstruction of the CT urogram is shown in Figure 2.

The right kidney showed a malrotated anteriorly faced duplex collecting system, with both ureters draining separately into the urinary bladder. The lower ureter crossed the midline and drained into the left vesicoureteric junction. A large calculus was seen in the right renal upper collecting pelvis, measuring 2.7 cm in maximum diameter and having a mean density of 1100 HU. It was associated with significant fat stranding and proximal moderate hydronephrosis. Another two nonobstructive calculi were noted in the kidney’s middle calyces, measuring 1.2 cm (mean density 896 HU) and 4.7 cm (mean density 464 HU), respectively. The right ureter was normal. A diagnosis of an ectopic kidney with a large calculus was made. The patient subsequently opted for percutaneous nephrolithotomy (PCNL) of the calculus.

Prior to the surgery, the interventional radiology department performed ultrasound-guided PCNL access under local anesthesia, with 20 cc of 1% xylocaine and a single 1-g dose of intravenous coamoxiclav antibiotic cover. An 18G needle was used to access the upper pole calyx of the upper moiety. The anatomy was confirmed using contrast injection and access to the upper pole calyx was achieved with J-wire. A 5-Fr sheath was parked in the renal pelvis; then, the renal system was dilated with contrast. A guidewire was negotiated across the pelvic calculus and into the urinary bladder. A 4-Fr catheter was then placed in the bladder. There were no immediate complications, and the patient was shifted to the operating room for double-J (DJ) stent placement under general anesthesia.

First, the patient was put in the lithotomy position and cystoscopy was performed. Once the right ureteric orifice was seen, a guidewire was passed along a 5-Fr open-ended ureteric catheter. Then, the patient was switched to the prone position. A super-stiff guidewire was passed into the renal pelvis, and dilatation was performed using 6, 7, 8, 9, 10, and 12-Fr dilators, followed by central rod placement, and five dilators were passed over the central rod and guidewire. A 24-Fr sheath was then passed through a 19.5-Fr nephroscope. A hard stone measuring 2.7 cm was seen. The stone was broken with a lithoclast over a period of three hours. After intracorporeal lithotripsy, the stone fragments were evacuated using multiple washes with upper system drainage. A DJ stent was placed along with a Foley catheter (nephrostomy), and a dry dressing was applied. No complications occurred during or after the surgery.

**Figure 3:**
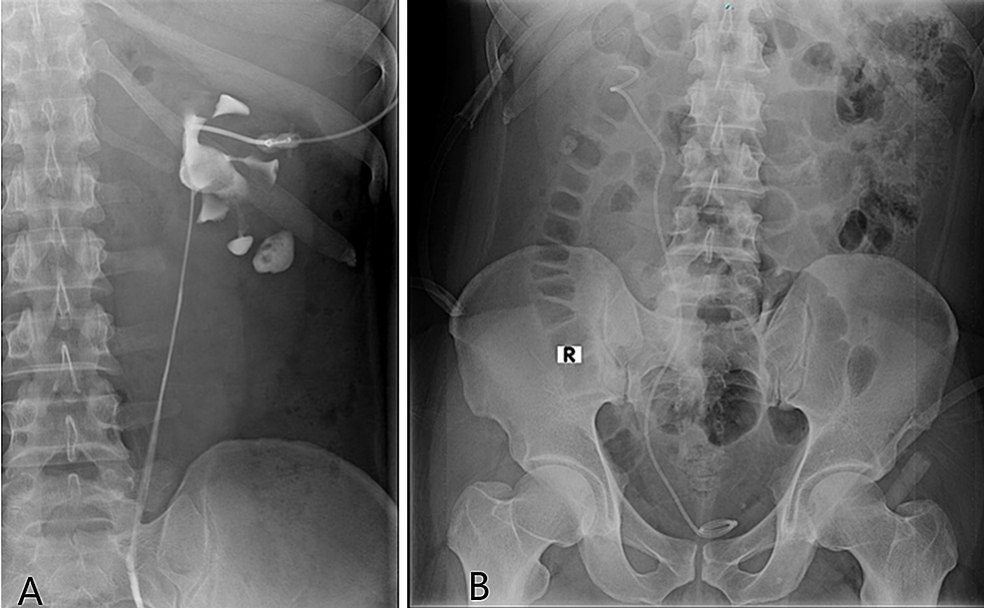
Image showing the CT urogram and kidney, ureter, and bladder X-ray. (A) Postop urogram showing the patient double-J stent in the upper calyx and patient ureter. (B) Postop kidney, ureter, and bladder X-ray showing DJ stent placement.

After recovering from the surgery, the patient was shifted to the ward and kept nil by mouth for six hours. He was started on intravenous normal saline at 120 ml per hour. Antibiotic coverage was supplied by a 2 g intravenous injection of cefoperazone twice daily for six days. Analgesia was provided with a 30-mg intravenous injection of ketorolac twice daily and a 4-mg intravenous injection of nalbuphine with dimenhydrinate three times a day. During his two-day stay in the ward, his vital signs and intake output fluids were recorded at one and six-hour intervals, respectively. A CT urogram and KUB X-ray were performed to check the position of the DJ stent (Figure 3A and 3B).

The nephrolithotomy tube was removed after two days, just prior to discharge. The patient was discharged on February 20, 2021, with prescribed analgesics, antibiotics, and potassium citrate tablets. A follow-up for stent removal was planned after one month, on March 19, 2021. After removing the DJ stent, an X-ray was taken and showed no signs of stones, strictures, or other abnormalities.

## Discussion

Renal fusion anomalies are defined as either partial or complete congenital fusion of the kidneys during the early embryonic period. Partial fusion anomalies include horseshoe kidney and CFRE, whereas complete fusion anomalies appear as a “cake” kidney, also known as fused pelvic kidney [6]. The four main types of crossed renal ectopia are unilateral crossed renal ectopia, crossed renal ectopia with fusion, crossed renal ectopia without fusion, and bilateral crossed renal ectopia. Nearly, 85-90% of cases of crossed renal ectopia are partially or completely fused, hence, the name CFRE. The six anatomical variants of CFRE are: (a) inferior CFRE, (b) sigmoid or S-shaped kidney, (c) lump kidney, (d) disc kidney, (e) L-shaped kidney, and (f) superior CFRE [7].

Crossed renal ectopia is more prevalent in males than in females, with a male/ female prevalence ratio of 3:2 [8]. It is a rare disease that goes mostly unnoticed throughout life. Typically, patients with crossed renal ectopia present with other abdominal diseases; consequently, the condition is mistaken for other diseases and treated accordingly unless an abdominal scan is performed, as was done in this study. The patient presented with right flank pain multiple times and was diagnosed with a renal stone without conducting any scans. Radiography plays an important part in diagnosing congenital anomalies; however, radiographic facilities are expensive and their availability is mostly limited to tertiary care hospitals. This is the main contributor to the underdiagnosis of such anomalies, as occurred in our patient, who went to local clinics when he experienced repeated instances of flank pain throughout his life. Renal fusion anomalies exhibit abnormalities of position (ectopia), migration, rotation, and vascular supply. The presence of such renal fusion anomalies poses difficulties and complications during abdominal, retroperitoneal, pelvic surgeries, renal transplantation, and interventional procedures.

Identifying CFRE is difficult, as its symptoms may be completely independent of the condition; therefore, it is mostly an incidental diagnosis that is found using various imaging techniques. Ultrasound is often used to detect CFRE due to its lower cost, lack of radiation, greater availability, and rapid results. However, intravenous pyelography (IVP) can also detect this condition; IVP clearly demonstrates the pelvicalyceal systems and the ureters and ureter from the ectopic kidney crossing the midline is diagnostic of this condition. CT scans and magnetic resonance imaging also provide a better understanding of the condition. The signs and symptoms typically presented by CFRE patients include urinary tract infection (UTI), stone formation, and generalized abdominal pain [9].

Several cases have been reported similar to the one in this study. A 2020 case report described a 19-year-old male patient with crossed complete fused left-to-right renal ectopia with a solitary left ureter [10]. The patient had reported symptoms of right flank pain for three months that did not compromise his daily routine. An ultrasound showed the absence of the left kidney in the left renal fossa and fused kidneys present in the right renal fossa. The patient was treated symptomatically and asked to return for routine follow-ups to rule out any complications. A descriptive analysis of six cases reported in India from 1997 to 2010 also concluded that crossed renal ectopia is mainly an incidental finding of ultrasound reports in patients presenting with flank pain [11]. The patients in the aforementioned study were children ranging from 14 months to 10 years of age. Three of the children had to undergo ureteric reimplantation. Of these, two underwent pyeloplasty for pelvic ureteric junction obstruction and one underwent nephrectomy. The study emphasized the importance of antenatal ultrasonography and follow-up, along with surgical intervention where needed.

CFRE can be difficult to manage due to the kidneys’ frequently abnormal shape, malrotation, and aberrant vasculature. These characteristics may lead to ureteropelvic junction obstruction and urinary tract diseases, such as vesicoureteral reflux, UTIs, ureteroceles, renal calculi, malignancy, and renovascular hypertension [12]. A case similar to ours was reported in 2013 at the Indira Gandhi Institute of Medical Sciences, India [13]. The patient had a similar reluctance toward the recommended urgent management and follow-up. He was likewise treated by placing a DJ stent in the crossed ectopic ureter and experienced no postoperative complications. Another study reported 22 patients with CFRE, which was discovered as an incidental finding [14]. These patients reported frequent complications; of the 22 patients, seven had hydronephrotic kidneys and three had nonfunctioning kidneys. Of the latter three patients, two were found to have a calculus obstruction. Vesicoureteral reflux was demonstrated in three of the five children reported in the study. The study also identified 11 patients with coarctation of the aorta, atrial septal defect, and anal atresia, therefore, emphasizing the need to examine and investigate other bodily systems in patients with CFRE.

Considering the rarity and potential complications associated with CFRE, the use of proper imaging techniques and immediate treatment along with follow-up should be emphasized and practiced.

## Conclusions

This study presented a case of an incidental diagnosis of CFRE in a 31-year-old patient, who presented with recurrent flank pain on the right side. We emphasize the timely use of ultrasound, not only in adulthood but also during the antenatal period, to exclude all chances of such abnormalities proceeding to the development of obstruction, infection, and neoplasia of the urinary system later in life. Patients diagnosed with CFRE should also be investigated for other developmental abnormalities. There are no specific guidelines for managing CFRE, and the kidney units do not need to be divided if they function normally. The treatment is mainly focused on symptomatic relief and changed as needed upon follow-up.
